# Saunders’s Medical Hand-Atlases

**Published:** 1901-09

**Authors:** 


Saunders's Medical Hand-Atlases. Atlas and Epitomé of Obstetric Diagnosis and Treatment. By Dr. O. Schaeffer, of Heidelberg. From the second revised German edition. Edited by J. Clifton Edgar, M.D., Professor of Obstetrics and Clinical Midwifery, Cornell University Medical School. With 122 colored figures on 56 plates, 38 other illustrations, and 317 pages of text. Philadelphia and London: W. B. Saunders & Co. 1901. [Cloth, $3.00 net.]

Saunders's Medical Hand-Atlases. Atlas and Epitomé of Labor and Operative Obstetrics. By Dr. O. Schaeffer, of Heidelberg. From the fifth revised German edition. Edited by Clifton J. Edgar, M.D., Professor of Obstetrics and Clinical Midwifery, Cornell University Medical School. With 14 lithographic plates, in colors, and 139 other illustrations. Philadelphia and London: W. B. Saunders & Co. 1901. [Cloth, $2.00 net.]

Here are two volumes of the Medical Hand-Atlas series which belong side by side, both from the pen of Dr. Oskar Schaeffer, of Heidelberg; edited by Dr. J. Clifton Edgar, of Cornell. The subject could hardly have been better presented by any author, while the work of editing the translation has been ably done by Dr. Edgar.

In the first work taken up, that on obstetric diagnosis and treatment, we have a really valuable book, and one which is of practical use. This is meant just as it is stated. It is not a figure of speech for the purpose of making a perfunctory review of one of a series of books which are all good in the departments of medicine they represent, nor is it said to be nice to a publisher or an author. That is not the Journal's style. The book is of value and a practical one, for the very simple reason that it imparts information of a practical character in a practical way and in such form as to be interesting and assimilable. The examiners of student papers on obstetrics, if they are not blinded by faith, know that in many cases the information on the subject has not been imparted—it has been forced into the mind of the soon to-be physician, in much the same manner as geese are prepared for the production of that expensive and indigestion-producing delicacy, pate de foie gras, by stuffing. A stuffed student is a sad object, and when he is stuffed with obstetrics he belongs in the danger class. That is why a book like this is of benefit to humanity. The first edition printed in the nineties was good; this is excellent. In its preparation Schaeffer has been "guided chiefly by the demands of the practical, clinical side of obstetrics."

How much better would many a new doctor be equipped to deliver a woman, had his professor of obstetrics had ideas like these of Schaeffer's: "I have therefore attempted to build up a practical system in the basis of my own experience. . . . An effort has been made to discuss the symptomatology with the greatest fulness compatible with brevity, instead of dismissing the subject with a few stereotyped phrases which are of no use in the individual case."

There is no known obstetrical condition or complication which is not referred to in this work and the illustrations are fitting accompaniments to the text. They are many and varied and by reason of the superior excellence of the plate and color work, are just about as near to good clinical instruction as one could very well expect in a book. There is nothing diagramatic about them. They are nearly all original reproductions in color of obstetric conditions. Those which are not color reproductions are most excellent drawings to scale, and present the idea in its most interesting form. The more one goes into the book the more pronounced is the feeling of confidence inspired by the nicely rounded sentences, the incisive style of the author.

The scope of this atlas may be better realised by a glance at its divisions. Part I., under the general heading of physiology and dietetics of pregnancy, labor and the puerperium, takes up physiology and diagnosis of pregnancy; anatomy, development and examination of the pelvis; normal labor; the puerperium and treatment of the new-born infant. Part II., dealing with pathology and treatment of pregnancy, labor and the puerperium, treats of the pathology of pregnancy, including abortion and premature labor; deformities of the pelvis and their influence on pregnancy and labor; pathology of labor; general remarks on exploration for purposes of diagnosis and treatment; pathology of the puerperium; prescriptions commonly used in obstetric practice. Truly, such a book will be useful not alone to students, but to a whole lot of general practitioners who apparently overlook or forget that there should be some difference in the method of delivering a woman and an automaton.

In the volume devoted to labor and operative obstetrics, Schaeffer has made a valuable addition to the literature of the subject. He not only goes into the work exhaustively and ably in the space at his command, but he has added much to the multitude of obstetric illustrations now in existence. These new representatives are not wholly diagramatic either, and therein lies their chiefest value. They show obstetric conditions and manipulations never very clearly represented on paper before. The necessity for this volume is very clearly shown by the editor in his preface when he says: "Obstetric surgery is for the most part performed, as it were, in the dark; whereas, the gynecologist, by means of improved amphitheaters, Trendelenburg posture, and electric light, can demonstrate to his audience each step in a given operation. He is enabled to choose his hour for operating, and everything, including the patient, is ready at the appointed time.

"On the contrary, a version, the correction of a malpresentation or malposition, the removal of an adherent placenta, usually demand that there shall be no delays."

In addition to the new illustrations referred to, the book has been brought up to the immediate present and the latest accepted views and methods, which have fulfilled the requirements of scientific proof, are presented, concisely, it is true, but in a form understandable and direct. This alone places the book in an enviable position, for it stands quite alone in this respect and even in its concise form is much more complete than many of the larger and more pretentious text-books—and it is much more useful too, as a helpful reference work.

To this edition, the fifth, Schaeffer has added a number of chapters, among them being the best methods of inducing premature labor and surgical obstetric operations. There is, too, a new idea presented—something quite unusual in obstetric work. Presentations have been divided into three classes instead of the usual two—favorable and unfavorable. To these perfunctory classifications, Schaeffer has added a third, the relatively favorable presentation. When one remembers that all these presentations are most excellently illustrated it does not take much to see how very necessary such a gem of obstetric literature is, not alone to the student but to the general practitioner as well.

The operative section is generally divided into purely obstetric operations on the fetus and ovum, and surgical obstetric operations on the mother. A further subdivision gives us: preliminary operations, operations during labor, and operations immediately following labor. In each subdivision the various methods are given in the order of their severity and the degree of danger involved.

There is a reason for this method for Schaeffer has found it most satisfactory in his teachings, and it more clearly emphasises the warning to the practitioner always to try the most conservative and least dangerous procedure first. The first part of the book deals with the act of parturition, considered from the standpoint of the practical obstetrician. In it are taken up positions of the fetus favorable for eutocia; presentations resulting from deformities of the pelvis or certain other typical conditions which may be specially favorable, or at least not unfavorable, for such conditions; positions and presentations which in themselves produce dystocia.

The second part concerns obstetric operations as follows: simple obstetric operations on the fetus and ovum, for correcting position, replacement of prolapsed parts; version; perforation and cranioclasis, embryotomy. Operation during labor includes expression, manual extraction, instrumental extraction.

Surgical obstetric operations include the induction of abortion and premature labor; dilating parturient canal ; delivery of the child through artificial passages; repair of lacerations and post partum operations.

The illustrations are almost beyond criticism. They are most admirably executed in color and show every obstetric condition and complication which could possibly be met with in practice. A careful study of the plates and drawings is the next best thing to actual clinical experience. They show every condition with a degree of naturalness which was quite unknown previous to the issue of these handy and valuable books of the medical hand-atlas series. Even if one is not particularly interested in the practice of obstetrics, the books should be owned for there is much material in them which bears on other sujects.

